# The Novel Monoacylglycerol Lipase Inhibitor MJN110 Suppresses Neuroinflammation, Normalizes Synaptic Composition and Improves Behavioral Performance in the Repetitive Traumatic Brain Injury Mouse Model

**DOI:** 10.3390/cells10123454

**Published:** 2021-12-08

**Authors:** Prabhuanand Selvaraj, Mikiei Tanaka, Jie Wen, Yumin Zhang

**Affiliations:** 1 Department of Anatomy, Physiology and Genetics, Uniformed Services University of the Health Sciences, 4301 Jones Bridge Road, Bethesda, MD 20814, USA; prabanan@gmail.com (P.S.); mikiei.tanaka.ctr@usuhs.edu (M.T.); jie.wen.ctr@usuhs.edu (J.W.); 2 Center for Neuroscience and Regenerative Medicine, Uniformed Services University of the Health Sciences, 4301 Jones Bridge Road, Bethesda, MD 20814, USA

**Keywords:** monoacylglycerol lipase inhibitor, 2-arachidonyl glycerol, glutamate receptors, GABA receptors, microglia, neuronal cell death, hippocampus, repetitive mTBI

## Abstract

Modulation of the endocannabinoid system has emerged as an effective approach for the treatment of many neurodegenerative and neuropsychological diseases. However, the underlying mechanisms are still uncertain. Using a repetitive mild traumatic brain injury (mTBI) mouse model, we found that there was an impairment in locomotor function and working memory within two weeks post-injury, and that treatment with MJN110, a novel inhibitor of the principal 2-arachidononyl glycerol (2-AG) hydrolytic enzyme monoacylglycerol lipase dose-dependently ameliorated those behavioral changes. Spatial learning and memory deficits examined by Morris water maze between three and four weeks post-TBI were also reversed in the drug treated animals. Administration of MJN110 selectively elevated the levels of 2-AG and reduced the production of arachidonic acid (AA) and prostaglandin E_2_ (PGE_2_) in the TBI mouse brain. The increased production of proinflammatory cytokines, accumulation of astrocytes and microglia in the TBI mouse ipsilateral cerebral cortex and hippocampus were significantly reduced by MJN110 treatment. Neuronal cell death was also attenuated in the drug treated animals. MJN110 treatment normalized the expression of the NMDA receptor subunits NR2A and NR2B, the AMPA receptor subunits GluR1 and GluR2, and the GABA_A_ receptor subunits α1, β2,3 and γ2, which were all reduced at 1, 2 and 4 weeks post-injury. The reduced inflammatory response and restored glutamate and GABA receptor expression likely contribute to the improved motor function, learning and memory in the MJN110 treated animals. The therapeutic effects of MJN110 were partially mediated by activation of CB1 and CB2 cannabinoid receptors and were eliminated when it was co-administered with DO34, a novel inhibitor of the 2-AG biosynthetic enzymes. Our results suggest that augmentation of the endogenous levels of 2-AG can be therapeutically useful in the treatment of TBI by suppressing neuroinflammation and maintaining the balance between excitatory and inhibitory neurotransmission.

## 1. Introduction

Traumatic brain injury (TBI) is one of the major causes of mortality and disability in modern industrialized societies [1], and has a significant impact on behavioral impairment, emotional disturbance and long term medical complications among the victims. It is estimated that there are nearly 1.7 million TBI cases each year in the United States, among which around 50,000 people died and 275,000 people were hospitalized [2]. Recently, attention has been focused on the repetitive mild traumatic brain injury (mTBI), due to its high incidence in military personnel deployed in the combat fields, and professional athletes in contact sports such as boxing and American football [3]. Similar to the civilian population, more than 80% of all TBI cases are classified as mTBI in the US military in the last two decades [4], and mTBI is thought to be a key risk factor for the late development of chronic traumatic encephalopathy and other neurodegenerative diseases [5]. Despite an increased understanding of the pathogenic mechanisms, there is still no FDA approved medication enabling functional recovery and therefore to develop novel therapeutic agents for TBI is urgently needed.

Modulation of the endocannabinoid system has emerged as an attractive strategy for many neurological and neuropsychiatric diseases [6,7]. The endocannabinoid system is composed of cannabinoid type 1 (CB1) and type 2 (CB2) receptors, endogenous ligands anandamide (AEA) and 2-arachidonoyglycerol (2-AG), and their biosynthetic and hydrolytic enzymes [8,9,10]. AEA is mainly synthesized from *N*-arachidonoyl phosphatidylethanolamine (NArPE) by *N*-acyl-phosphatidylethanolamine phospholipase D (NAPE-PLD) and degraded by fatty acid amide hydrolase (FAAH) [11]. 2-AG is produced from the membrane phosphatidylinositol-4, 5-bisphosphate by activation of phospholipase C and diacylglycerol lipases (DAGLα and DAGLβ), and hydrolyzed primarily by monoacylglycerol lipase (MAGL) [12]. Several studies from our group and others have demonstrated that augmentation of AEA and 2-AG by inhibition of their hydrolytic enzymes is protective in the animal models of TBI [13,14,15,16,17,18,19]. Consistent with the findings that administration of 2-AG attenuates neurological deficits, neuroinflammation, neurovascular dysfunction and excitotoxicity in the rodent model of closed head injury [20,21,22], enhancement of the endogenous levels of 2-AG by inhibition of MAGL has also been shown to ameliorate synaptic dysfunction, cognitive decline and long-term neurodegeneration in mice suffering from both single and repetitive TBI [15,16]. Inhibition of MAGL might be more beneficial than the exogenously administered 2-AG given the context-dependent and region-specific nature of the 2-AG produced endogenously. Recently, MAGL is shown to be the principal enzyme controlling the production of arachidonic acid and proinflammatory prostaglandins in the brain [23]. However, it remains unclear whether the therapeutic effects of the inhibition of MAGL are due to activation of the CB1 and CB2 cannabinoid receptors and/or the reduction of the eicosanoid signaling.

MJN110 is a recently developed MAGL inhibitor and was shown to have improved selectivity, potency and fewer side effects compared to the previously available MAGL inhibitors [24,25]. Administration of MJN110 has been shown to have profound effects to reduce stress, anxiety, neurodegeneration and to alleviate both inflammatory and neuropathic pain [25,26,27,28]. In this study, we found that post-injury treatment with MJN110 improved locomotor function, spatial learning and memory, suppressed neuroinflammation, reduced neuronal death and normalized the expression of glutamate and GABA receptor subunits in cerebral cortex and hippocampus in the repetitive mTBI mouse model. Our data suggested that the therapeutic effect of MJN110 is partially mediated by activation of CB1 and CB2 cannabinoid receptors and is dampened by blockade of the 2-AG biosynthesis.

## 2. Materials and Methods

### 2.1. Chemicals

The DAGLα and β inhibitor DO34 was generously donated by Dr. van der Stelt (Leiden University, Leiden, The Netherlands) and also purchased from Aobious Inc. (Gloucester, MA, USA), The MAGL inhibitor MJN110, the CB1 receptor (CB1R) antagonist AM281, the CB2R antagonist AM630, and the deuterated AEA, 2-AG and arachidonic acid (AEA-d4, 2-AG-d5 and AA-d8) were purchased from Cayman Chemicals (Ann Arbor, MI, USA). 

### 2.2. Animal Model and Drug Treatment

Male, 8–12 weeks old C57BL/6J mice were purchased from the Jackson Laboratory (Bar Harbor, ME, USA). All animal procedures were performed in accordance with the guidelines established by the National institutes of Health (NIH, Bethesda, MD, USA) and approved by the Uniformed Services University Institutional Animal Care and Use Committee. Repetitive closed head injury was performed in mice using an electromagnetic controlled stereotaxic impact device as we described previously [18]. Briefly mice were anesthetized with 3% isoflurane prior to stabilizing the head using ear bars in a mouse stereotaxic frame (Stoelting Co., Wood Dale, IL, USA). A midline sagittal incision was made to expose the skull. The stereotaxic electromagnetic impactor with a 3.0 mm steel tip impounder was used to deliver a single controlled cortical impact, delivered at coordinates of 1.8 mm caudal to bregma and 2.0 mm left to midline with a controlled velocity at 3.0 m/s, an impact depth of 2.0 mm and a dwell time at 100 milliseconds. Mice with depressed skull fracture or visible hemorrhage were excluded from the study. Sham mice underwent identical surgical procedures as the injured group, but no impact was delivered. After impact, the skin was sutured and the animals were allowed to recover from anesthesia and then returned to their home cages. A second and third identical closed head injury procedures were performed on day 2 and day 3 after the initial injury. The skull fracture rate was less than 2.0% during the surgical procedures.

For drug treatment, mice were given an intraperitoneal injection of various doses of MJN110 (0.5, 1 and 2.5 mg/kg), a selective inhibitor of MAGL, at 30 min after each impact and then once a day for 5 additional days (eight injections in total). At the selected concentrations, MJN110 has been shown to dose-dependently increase the brain levels of 2-AG and alleviate neuropathic pain in mice [24,25]. To determine the cannabinoid receptor dependency, MJN110 (2.5 mg/kg) was co-administered with the CB1R antagonist AM281 (3 mg/kg) and the CB2R antagonist AM630 (3 mg/kg), respectively. To examine the importance of 2-AG molecule in this TBI mouse model, MJN110 was co-administered with DO34 (30 mg/kg), a selective inhibitor of the 2-AG synthetic enzymes diacylglycerol lipase alpha and beta (DAGLα and β) to block the 2-AG synthesis [29]. The individual or combined pharmacological inhibitors were dissolved in 1:1:18 ratio of DMSO-cremophor-saline that served as a vehicle control.

### 2.3. Behavioral Assay

**Figure 1:**
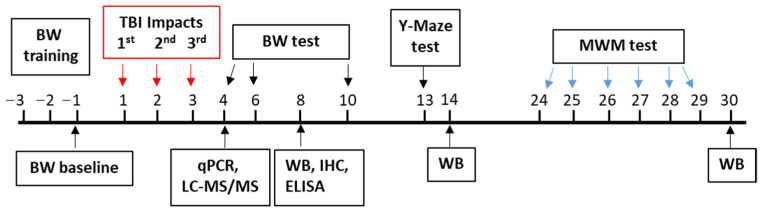
Schematic diagram of the experimental designs. The red arrows represent the days when the first, second, and third TBI was delivered. The repetitive closed head injury was established using an electromagnetic controlled stereotaxic impact device. BW, beam walk; qPCR, quantitative polymerase chain reaction; LC-MS/MS, liquid chromatography with tandem mass spectrometry; WB, western blot; IHC, immunohistochemistry; ELISA, enzyme-linked immunoassay; MWM, Morris water maze.

The beam-walk, Y-maze and Morris water maze were performed at various time points post-injury to assess fine motor movement [13,18,30], working memory [13,18] and spatial learning and memory [18,31]. The timeline for all the behavioral tests, and molecular, biochemical and immunohistochemistry assessments is illustrated in Figure 1.

#### 2.3.1. Beam Walk Test

The beam-walk test was performed to assess fine motor movement as we previously described [13]. Briefly, the beam-walk apparatus consists of a wooden beam measuring 6 mm in width, 120 cm in length, and suspended 30 cm above a table. Mice were trained to walk on the beam for 3 days before surgery and at the end of the third day the baseline values (i.e., the number of foot faults per 50 steps) were collected. The beam-walk balance test was performed at days 4, 6 and 10 after the initial impact and the number of foot faults per 50 steps was recorded. 

#### 2.3.2. Spontaneous Alternation Y-Maze Test 

At 13 days after repetitive closed head injury, mice were tested in a Y-maze apparatus. The Y-maze device (Stoelting Co., Wood Dale, IL, USA) has a symmetrical plastic Y shape with arms measuring 25 cm long, 8 cm wide and 15 cm high. Mice were placed at the end of a randomly chosen arm as a starting point, and allowed to explore the maze for 5 min. Each arm visit was counted as a mouse moving all four paws into the arm, and each alternation was defined as a consecutive entry into three different arms. The sequence and the number of entries to each arm were video recorded. The percentage alternation was calculated based on the formula: total number of alternations/(total number of arm entries-2) × 100 as previously described [13,14]. The spontaneous alternation Y-maze test measures hippocampus-based working memory [32], and was used to evaluate the effect of drug treatment on TBI-induced working memory deficits. 

#### 2.3.3. Morris Water Maze Test

The Morris water maze test was performed at 24–29 days post-TBI to examine spatial learning and memory as described previously with some modifications [31]. Briefly, a circular water tank with a diameter of 1.5 m was used to perform the Morris water maze. For extra-maze visual cues, the four walls around the tank were permanently hanged with different colors and shapes for orientation. A round transparent platform (with a diameter of 11.5 cm) was hidden 1 cm beneath the surface of the water at the center of a given quadrant of the water tank. Mouse received training in the Morris water maze for 5 days and each session was composed of four trials, plus a probe trial 24 h after the last hidden platform test. For each trial, the mouse was released facing the wall of the tank and allowed to search, find, and stand on the platform for 15 s within the 60-s trial period. The time it took to find the platform was calculated as Latency. In each training session, the starting quadrant point and sequence of the four quadrants from where the mouse was released into the tank were randomly chosen so that it was varied among the separate sessions for each animal and was different for individual animals. In the latter case, the mouse who couldn’t find the platform was guided to the platform and remained there for 15 seconds before being returned to the home cage. The probe trial was conducted by removing the platform and releasing the mouse facing the wall exactly from the opposite or parallel side where the platform was hidden previously. The task performance was recorded for 60 s. Tracking of animal movement was achieved with the ANY-maze video-tracking system.

### 2.4. Quantitative Real-Time PCR 

Mice were sacrificed on day 4 after three impacts to extract total RNA from the ipsilateral cortex using TRIzol (Sigma, St Louis, MO, USA) following the manufacturer’s instructions. RNA yield and purity were evaluated using a NanoDrop spectrophotometer. RNA (1 μg) was reverse transcribed to cDNA. First-strand cDNA was synthesized with Maxima First Strand cDNA Synthesis Kit (Thermo Fisher Scientific, Waltham, MA, USA). Amplified cDNAs were diluted at 1:10 in ultrapure water and subjected to SYBER green master mix (Applied Biosystems, Grand Island, NY, USA) based real-time PCR on a light cycler 480 II Roche System with primers IL-1β, IL-6, TNF-α, iNOS, CB1R, CB2R, COX1, COX2, and GAPDH. PCR reactions were conducted as follows: 95 °C for 15 s, 75 °C for 1 min, and 60 °C for 30 s for a total of 40 cycles, followed by a melting point determination or dissociation curves. All samples were run in triplicate. Relative levels of gene expression were determined by the 2^−ΔΔCt^ method normalized to the expression of GAPDH. 

### 2.5. PGE_2_ Assay

For PGE_2_ assay, the ipsilateral cortex was isolated from day 4 post-TBI and subjected to homogenization with 40 μL of 0.02% trifluoroacetic acid (TFA) and 100 μL of acetonitrile on ice. To extract maximal lipid, 140 μL of tissue homogenate was dispersed in 1 mL acetonitrile by vortex and left at 4 °C overnight. On the next day the homogenate-acetonitrile mixture was centrifuged at 2000× *g* for 5 min to remove the debris and the supernatant was transferred to a silanized glass tube. The supernatant was evaporated under the nitrogen gas streaming in a water bath (approx. 35 °C) and then reconstituted with acetonitrile. The levels of PGE_2_ in the lipid extract were measured using PGE_2_ enzyme-linked immunoassay (ELISA) kit following the manufacturer’s protocol (Cayman Chemical, Ann Arbor, MI, USA).

### 2.6. Western Blot

To determine the levels of phosphorylated ERK, phosphorylated AKT, NMDA and AMPA receptor subunits, GABA_A_ subunits, GAD67, VGLUT2, COX1 and COX2, mice were sacrificed at 8, 14 and 30 days post-TBI. The ipsilateral cortical tissues adjacent to the site of injury were removed and homogenized in RIPA buffer supplemented with 1x protease & phosphatase inhibitor cocktail (Cat# 1861280, Thermo Fisher Scientific, Rockford, IL, USA). Protein concentration was determined for each sample, and equal amount of proteins were run on 4–15% SDS-PAGE (Bio-Rad). Thereafter, proteins were transferred onto nitrocellulose membranes, blocked for 1 h with 5% BSA at room temperature, and then incubated overnight at 4 °C with the primary antibodies. These antibodies included anti-rabbit phosphorylated (p-) p44/42 MAPK (T202/Y204) (1:3000; cat# 9101S, Cell Signaling, Danvers, MA, USA), anti-rabbit p-AKT (S473) (1:2500; cat# 9271S, Cell Signaling), anti-mouse COX1 (1:500; cat#160109, Cayman Chemical), anti-mouse COX2 (1:500; cat#160109, Cayman Chemical), anti-Rabbit NR2A (1:1000; cat# 07-632, Millipore, Burlington, MA, USA), anti-Rabbit NR2B (1:1000; cat# AB1557P, Millipore), anti-Rabbit GluR1 clone C3T (1:1000; cat# 04-855, Millipore), anti-rabbit ionotropic glutamate receptor 2 (1:4000; cat# ab20673, Abcam, Cambridge, MA, USA), anti-mouse VGLUT2 (1:2500; cat# MAB5504, Millipore), anti-NMDAR1 (1:2000; cat# ab109182, Abcam), anti-mouse GAD67 clone 1G102 (1:4000; cat# MAB5406, Millipore), anti-mouse GABA_A_ receptor α1 protein clone N95/35 (1:2500; cat# MABN489, Millipore), anti-mouse GABA_A_ receptor β2,3 chain clone BD17 (1:2500; cat# MAB341, Millipore) and anti-mouse GABA_A_ γ2 subunit clone KC4-8A7 (1:2500; cat# MABN875, Millipore). After probing with a goat anti-rabbit or anti-mouse IgG (H + L)-HRP conjugated secondary antibody (1:2500; Bio-Rad, Hercules, CA, USA) for 1 h at 25 °C, protein bands were detected using the Supersignal West Pico Chemiluminescence (Thermo Fisher Scientific, Waltham, MA, USA). Membranes were subsequently probed for the expression of β-actin as a loading control. Images were acquired with a Chemidoc Touch image system (BioRad) and analyzed using ImageJ software.

### 2.7. LC-MS/MS Analysis 

The cortical tissues isolated from day 4 TBI mice were subjected to liquid chromatography coupled with tandem mass spectrometry (LC-MS/MS) for the measurement of 2-AG, AEA and AA. The tissue was homogenized with 40 μL of 0.02% TFA, 250 μL of acetonitrile, and 250 picomoles of 2-AG-d5 and AA-d8 and 0.5 picomoles of AEA-d4 (Cayman Chemical) using a Potter homogenizer at 4 °C. The homogenate was dissolved completely in 2.5 mL acetonitrile by vortex and kept at 4 °C overnight. The homogenate was subjected to centrifugation at 2000× *g* for 5 min to remove the debris, and then the supernatant was evaporated under nitrogen gas streaming in a water bath (approx. 35 °C). The lipid was re-suspended with 100 μL of acetonitrile and stored at −80 °C until use.

An HPLC system (1200 Series, Agilent Technologies, Santa Clara, CA, USA) was used with a reverse phase guard column (Wide Pore C18 (ODS), 4 × 2 mm ID; Phenomenex, Torrance, CA, USA), and the column (Sephasil Peptide C18, 5 μ, ST, 100 × 4.6 mm ID; Pharmacia Biotech, Piscataway, NJ, USA) was maintained at 40 °C. The mobile phase was composed of solvent A (0.2% formic acid in water) and solvent B (0.2% formic acid in methanol), and the following gradient was used: 62% A/38% B isocratic for 30 s, ramp to 90% B in 60 s, isocratic at 90% B for 18.5 min, ramp back to 62% A/38% B in 60 s, and re-equilibrate at 62% A/38% B for 8 min. The flow rate was 0.4 mL/min. The HPLC output was directed into the TurboV electrospray ionization (ESI) source of a Q-Trap 4000 mass spectrometer (AB Sciex, Framingham, MA, USA). The injection volume was 20 μL. LC-MS/MS analysis was performed in a positive mode with the ion source temperature of 600 °C, a spray voltage of 5.5 kV, and a declustering potential of 45 V. Multiple reactions monitoring (MRM) was performed on the transitions *m*/*z* 379→287 for 2-AG, 348→62 for AEA, and 304→121 for AA. The concentrations of 2-AG, AEA and AA were determined by calculating the corresponding peak area ratio to the internal standard (IS) using a linear fit weighting to the calibration curve.

### 2.8. Immunohistochemistry

On day 8 post-TBI, mice were anesthetized using a combination of ketamine and xylazine (90 mg/kg ketamine/10 mg/kg xylazine in a volume of 10 μL/g body weight, i.p.) and then intracardially perfused with ice-cold PBS followed by 4% paraformaldehyde. The brains were quickly removed from the skulls and post-fixed in 4% paraformaldehyde at 4 °C overnight. On the following day, brains were cryoprotected in 30% sucrose in PBS at 4 °C until sinking, embedded in Tissue Tek OCT (Sakura Finetek, Torrance, CA, USA) and stored at −80 °C until use. Coronal sections of the brain were cut at 30 μm using SM2000R microtome (Leica, Buffalo Grove, IL, USA) and the series sections (10 to 12 slices) were collected in a 24-well plate and stored in cryoprotectant solution at −20 °C until the assay for immunostaining. Primary antibodies used for immunostaining included the anti-goat Iba1 (1:300; Cat# ab48004, Abcam), and anti-mouse GFAP (1:500; Cat# 3670, Cell Signaling) antibodies that were used to determine whether there was an altered expression of microglia and astrocytes, respectively. Free floating sections were immunostained using the specific antibodies listed above, followed by incubation with the corresponding fluorescent-labeled secondary antibodies. The sections were washed with 1× PBS twice, mounted on slides and then covered with fluoroshield mounting medium with DAPI. Immunofluorescence images were obtained with a fluorescence microscope (Eclipse TE-2000U, Nikon, Melville, NY, USA). The cells with both DAPI and expected fluorescence were defined as positively stained cells. Negative controls were routinely performed in which the primary antibodies were omitted. All immunofluorescence data were obtained in a minimum of 5–7 serial sections from the brain tissues of each animal. GFAP and Iba1 immunostaining images were taken, analyzed and quantified using the NIH ImageJ software.

### 2.9. Fluoro-Jade C Staining

Fluoro-Jade C (FJC) staining was applied to discern the degenerated neurons. Free floating sections mounted on slides were incubated in the solution with FJC (0.0004% solution, EMD Millipore, Temecula, CA, USA) and 4–6-diamidino-2-phenylindole (0.5 μg/mL) for 20 min, followed by 3 × 1 min wash with distilled water. The slices were dried naturally at room temperature without light. The images were taken using a fluorescence microscope (Nikon Eclipse TE-2000U) and the FJC positive cells were counted and expressed as mean cell numbers per mm^2^.

### 2.10. Statistical Analysis

Statistical analysis was performed using Prism 7 software (GraphPad Software, Inc., San Diego, CA, USA). For multiple comparison, one-way or two-way analysis of variance (ANOVA) followed by Tukey-Kramer’s post-hoc tests were used. Differences in significance were defined at *p* < 0.05 and data were expressed as means ± SEM.

## 3. Results

### 3.1. Locomotor and Working Memory Deficits Associated with Repetitive mTBI Were Mitigated by the MAGL Inhibition

**Figure 2:**
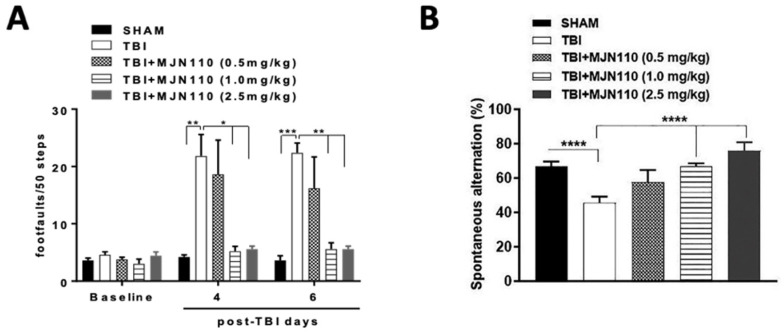
MJN110 treatment attenuated TBI induced impairment in locomotor function and working memory. (**A**) Effect of MJN110 on fine motor movement was assessed by beam-walk test, and the deficits in fine motor movements were recorded as foot faults on day 4 and day 6 in post-TBI animals. (* *p* < 0.05, ** *p* < 0.01, and *** *p* < 0.001; *n* = 10 animals/group) (**B**) Spontaneous alternation Y maze test was performed on day 13 post-injury. (**** *p* < 0.0001; *n* = 10 animals/group). Bars represent standard error of the mean (mean ± SEM).

To determine the therapeutic effect of augmentation of 2-AG through pharmacological inhibition of its hydrolysis, different doses of MJN110 (0.5, 1.0 and 2.5 mg/kg) were given intraperitoneally at 30 min after each impact and then once daily for 5 additional days. Treatment with MJN110 at 1.0 and 2.5 mg/kg, but not at 0.5 mg/kg significantly reduced the number of foot faults in beam-walk test at 4 and 6 days post-TBI (Figure 2A). The number of spontaneous alternations in the Y-maze was also significantly reduced on day 13 post-TBI and increased by MJN110 treatment (Figure 2B). Administration of MJN110 to the sham animals did not impact the performance in both beam-walk and Y-maze tests (data not shown). Since MJN110 at 2.5 mg/kg had greater therapeutic effects to mitigate TBI-induced locomotor and working memory deficits, this dose was mostly used for the subsequent studies.

### 3.2. MAGL Inhibition Selectively Elevated the Endogenous 2-AG Levels in the TBI Mouse Brain

**Figure 3:**
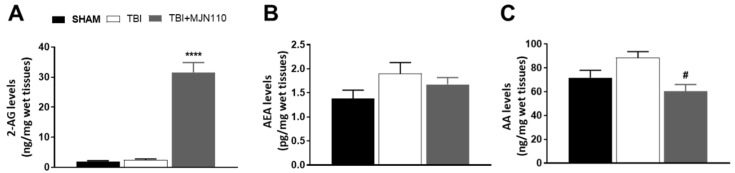
Administration of MJN110 elevated the brain levels of 2-AG and reduced the production of AA in TBI mouse brain. Fresh ipsilateral mouse cortical tissues on day 4 post-TBI were subjected to LC-MS/MS analysis. (**A**) 2-AG levels were significantly increased in the MJN110 (2.5 mg/kg)-treated group compared to the sham and TBI/vehicle groups (**** *p* < 0.0001). (**B**,**C**) Levels of AEA and AA were examined by LC-MS/MS, and the production of AA was reduced by MJN110 treatment (# *p* < 0.05 vs. TBI/vehicle group). (*n* = 5 animals in each group; bars represent means ± SEM).

To determine the selectivity of MJN110 on 2-AG metabolism in the TBI mouse brain, the levels of 2-AG, AEA and AA in the ipsilateral cortex at 4 days post-TBI were examined using LC-MS/MS. Although there were no differences in the brain levels of 2-AG, AEA and AA between the TBI and sham control animals (Figure 3A–C), administration of MJN110 (2.5 mg/kg) selectively elevated the brain levels of 2-AG (Figure 3A), but not AEA (Figure 3B). The levels of AA were significantly reduced in the MJN110 treatment group (Figure 3C). These results further support the notion that MAGL is a principal enzyme to regulate AA synthesis in the brain [29].

### 3.3. Inhibition of MAGL Attenuated TBI-Induced Neuroinflammation and Neuronal Cell Death

**Figure 4:**
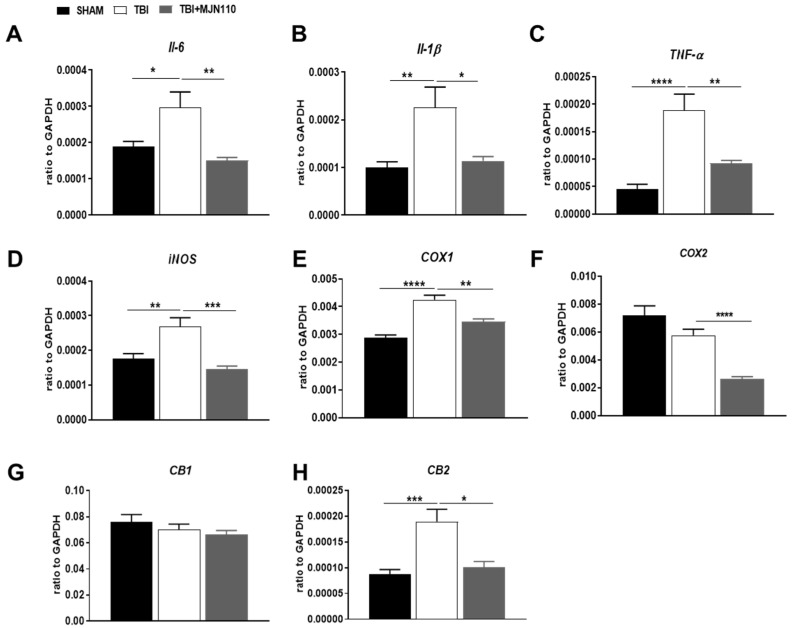
Treatment with MJN110 suppressed the expression of neuroinflammatory molecules and the CB2 receptor in the TBI mouse brain. The fresh cortex tissues from mice 4 days post-TBI were subjected to qRT-PCR analysis. The mRNA expression of pro-inflammatory cytokines IL-6, IL-1β and TNF-α (**A**–**C**) and the expression of iNOS, COX-1, COX-2, CB1 and CB2 receptors (**D**–**H**) were compared between sham, TBI/vehicle and the MJN110 (2.5 mg/kg) treatment groups. (* *p* < 0.05, ** *p* < 0.01, *** *p* < 0.001 and **** *p* < 0.0001. *n* = 15 animals in each group; Bars represent means ± SEM).

**Figure 5:**
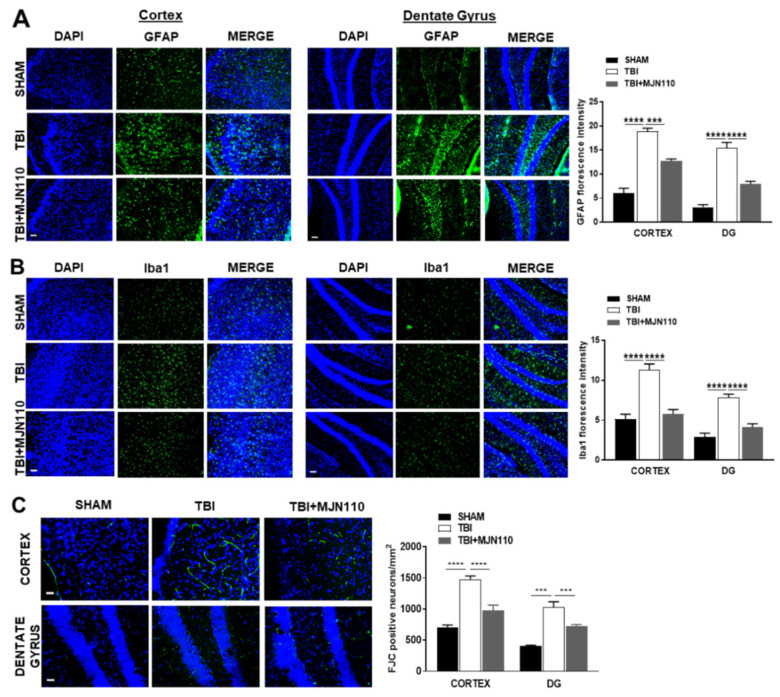
MJN110 treatment reduced glial cell accumulation and neuronal death in the ipsilateral cortex and dentate gyrus of TBI mouse brain. At 8 days post-injury, the (**A**) GFAP-, (**B**) Iba1- and (**C**) FJC-positive immunostainings were significantly increased in the ipsilateral cortex and dentate gyrus (DG) of the TBI mouse brain, and these changes were attenuated by MJN110 (2.5 mg/kg) treatment. (*** *p* < 0.001 and **** *p* < 0.0001; *n* = 3 animals in each group. Bars represent means ± SEM, and the scale bar is equal to 50 μm). DAPI, 4′,6-diamidino-2-phenylindole; GFAP, glial fibrillary acidic protein; FJC, Fluoro-Jade C.

TBI is commonly associated with enhanced inflammatory response which is characterized by activation of glial cells, infiltration of peripheral leukocytes and release of a variety of inflammatory mediators including cytokines and chemokines [33]. It is possible that such changes in cellular and molecular events can initiate neuropathological process in the animal model of TBI. We measured the mRNA levels of several pro-inflammatory mediators and the expression of CB1 and CB2 cannabinoid receptors. Quantitative RT-PCR analysis showed that the mRNA expression of IL-6, IL-1β, TNFα, iNOS and COX-1 was significantly increased in the ipsilateral cortices of TBI mice and all the changes were reversed by MJN110 (2.5 mg/kg) treatment (Figure 4A–E). However, the expression of COX-2 was not altered between the sham and TBI groups, though a significant reduction was found in the MJN110 treated group (Figure 4F). There was no difference in the levels of CB1 receptor among the sham, TBI/vehicle and treatment groups (Figure 4G), whereas the expression of CB2 receptor was significantly elevated in the TBI/vehicle and reduced by MJN110 treatment (Figure 4H). In harmony with these findings, immunostaining using GFAP and Iba1 revealed that the accumulation of astrocytes and microglia in the ipsilateral cortex and dentate gyrus of the TBI mouse brain was significantly reduced by MJN110 treatment (Figure 5A,B). Neuronal cell death in the TBI mouse ipsilateral cortex and hippocampal dentate gyrus examined by Fluoro-Jade C staining was also reduced in the MJN110 treated animals (Figure 5C).

### 3.4. Alteration of the Ionotropic Glutamate and GABA Receptor Components and the Survival Signaling Molecules in the TBI Mouse Hippocampus Was Restored by MAGL Inhibition

**Figure 6:**
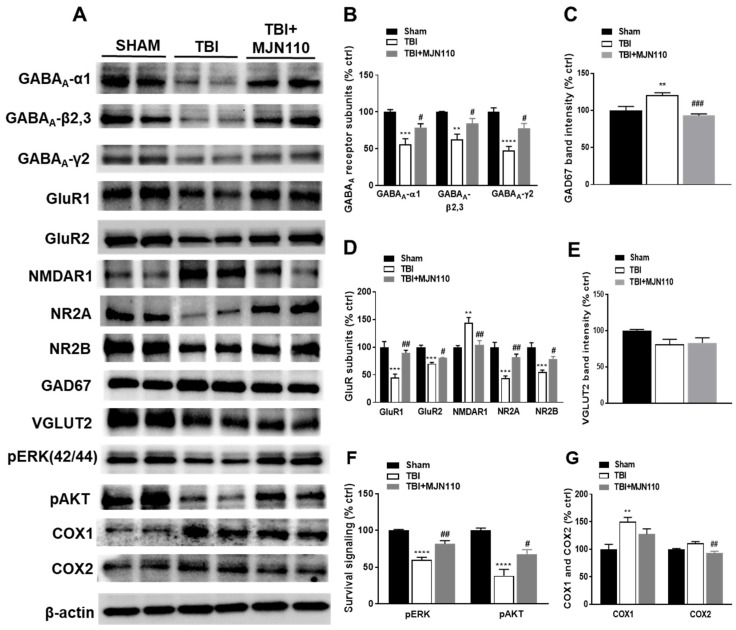
MJN110 treatment restored the altered expression of the ionotropic glutamate and GABA receptor components and the survival signaling molecules in the TBI mouse hippocampus. At 8 days post-TBI, the expression of GABA_A_-α1, GABA_A_-β2,3, GABA_A_-γ2, GluR1, GluR2, NR2A, NR2B, pERK and pAKT in the ipsilateral hippocampal tissues was significantly reduced in the TBI vehicle group, and reversed by the MJN110 (2.5 mg/kg) treatment (**A**,**B**,**D**,**F**). The expression of GAD67 and NMDAR1 was upregulated in the TBI/vehicle group and reduced by the MJN110 treatment (**A**,**C**,**D**). There were no changes in the expression of VGLUT2 in all the experimental groups (**E**). The expression of COX-1, but not COX-2 was increased in the TBI/vehicle group (**G**). (** *p* < 0.01, *** *p* < 0.001, and **** *p* < 0.0001 comparison between sham and TBI/vehicle; # *p* < 0.05, ## *p* < 0.01 and ### *p* < 0.001 comparing TBI/vehicle vs. TBI + MJN110 groups. *n* = 5 animals in each group). Bars represent means ± SEM).

TBI-induced cognitive deficits can be caused by the loss of hippocampal neurons and the alteration of receptor molecules involved in the process of learning and memory such as GABA and glutamate receptors [34]. Using hippocampal tissue lysates, we examined the expression of glutamate and GABA receptor subunits at 8 days post-TBI. The expression of GABA_A_ receptor subunits α1, β2, 3 and γ2 (Figure 6A,B) and glutamate receptor subunits GluR1, GluR2, NR2A and NR2B (Figure 6A,D) were significantly reduced in the TBI vehicle group, and these changes were restored in the MJN110 (2.5 mg/kg) treated animals. Conversely, NMDAR1 (Figure 6D) and the GABA synthetic enzyme glutamate decarboxylase (GAD) 67 (Figure 6C) were increased in the TBI/vehicle and reduced by MJN110 treatment. Notably, there was no difference in the expression of the vesicular glutamate transporter 2 (VGLUT2) in all the experimental groups (Figure 6E). The phosphorylation of ERK and AKT (Figure 6F) were significantly down regulated in the TBI mouse brain and reversed by MJN110 treatment. Similar to the changes in the mRNA expression, Western blot also revealed the increased expression of COX-1, but not COX-2 in the TBI mouse hippocampal tissues, and treatment with MJN110 attenuated the expression of both enzymes, although only the expression of COX-2 was significantly reduced when compared to the TBI/vehicle group (Figure 6G).

### 3.5. Reversal of Locomotor and Short-Term Working Memory, but Not Long-Term Cognitive Function by MJN110 Was Mediated by the CB1 Receptor Activation

**Figure 7:**
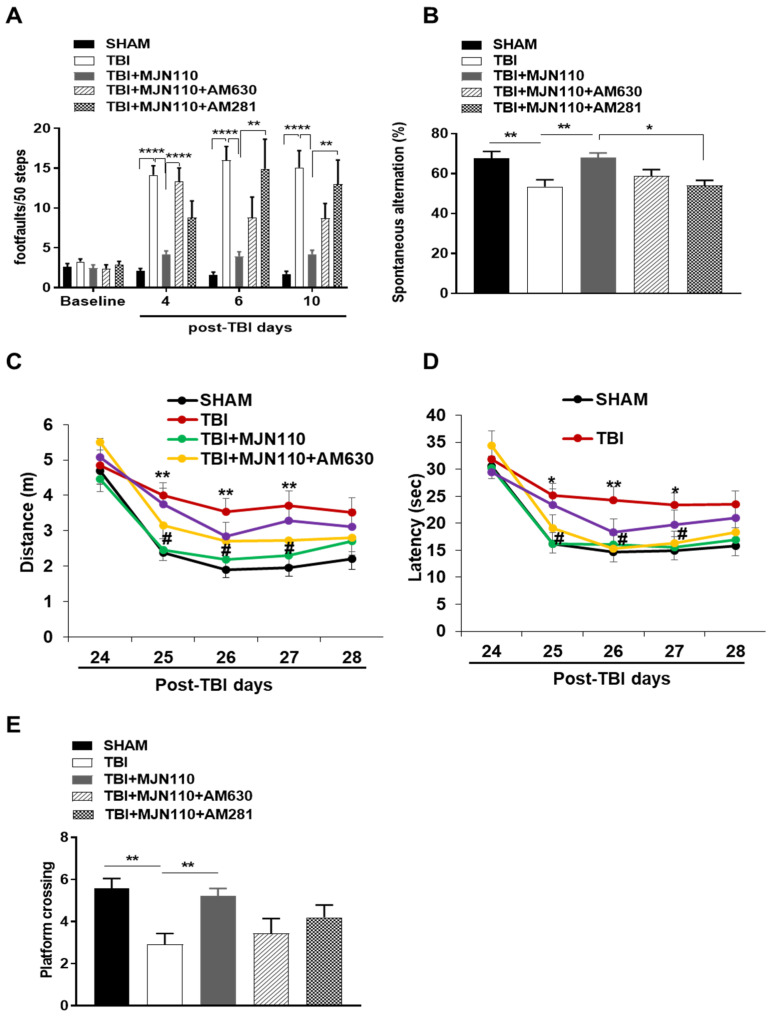
TBI-induced motor and working memory deficits, but not the alteration of spatial learning and memory were attenuated by MJN110 through cannabinoid receptor activation. (**A**) In Beam walk test, the number of foot faults per 50 steps was assessed at 4, 6 and 10 days post-TBI. (** *p* < 0.01 and **** *p* < 0.0001. *n* = 10 animals in each group). (**B**) On day 13 post injury, spontaneous alternation in Y maze test was examined (* *p* < 0.05, and ** *p* < 0.01). (**C**,**D**) Morris water maze test was conducted to assess spatial learning at 24–28 days post-TBI (* *p* < 0.05 and ** *p* < 0.01 comparing sham vs. TBI groups; # *p* < 0.05 comparing TBI vs. TBI + MJN110 (2.5 mg/kg) groups), and (**E**) the probe trial to assess memory was performed on day 29 post-TBI. (** *p* < 0.01). *n* = 10 animals in each group; Bars represent means ± SEM.

To determine whether the action of MJN110 is mediated through cannabinoid receptors, we administered MJN110 together with the CB1 receptor antagonist AM281 or the CB2 receptor antagonist AM630 in mice undergoing repetitive mTBI. On day 4 post-injury, the locomotor impairment was significantly reduced in the MJN110 (2.5 mg/kg) treated animals, and the therapeutic effect of MJN110 was greatly diminished in the AM630 (3 mg/kg) co-treatment group. Despite that treatment with AM281 (3 mg/kg) also led to increased numbers of foot faults, there was no significant difference when it was compared with the MJN110 treatment alone (Figure 7A). Interestingly at 6 and 10 days post-TBI, the locomotor deficits were completely reversed by co-treatment with AM281 (Figure 7A). Similarly, the improvement of MJN110 on spontaneous alternation in Y-maze at 13 days post-TBI was also significantly reversed by AM281 *(*Figure 7B), suggesting the major contribution of CB1 receptor in improving working memory. Next, we performed Morris water maze to examine spatial learning and memory at 24–29 days post-TBI (Figure 7C–E). TBI animals travelled longer distance and spent extended latency to find the submerged platform at training days 25–28 compared to the sham animals. MJN110 treatment significantly reduced the travel distance and latency compared to the TBI/vehicle group (Figure 7C,D). Similarly, in the probe trial on day 29 post-TBI, TBI animals had fewer entries to the hidden platform than the sham and MJN110 treated animals (Figure 7E). The effect of MJN110 was not significantly altered by co-administration of either AM281 or AM630, despite a tendency of improvement in the AM281 treated animals (Figure 7C–E). 

### 3.6. Inhibition of MAGL Normalized the Expression of Ionotropic Glutamate and GABA Receptors at 30 Days Post-TBI

**Figure 8:**
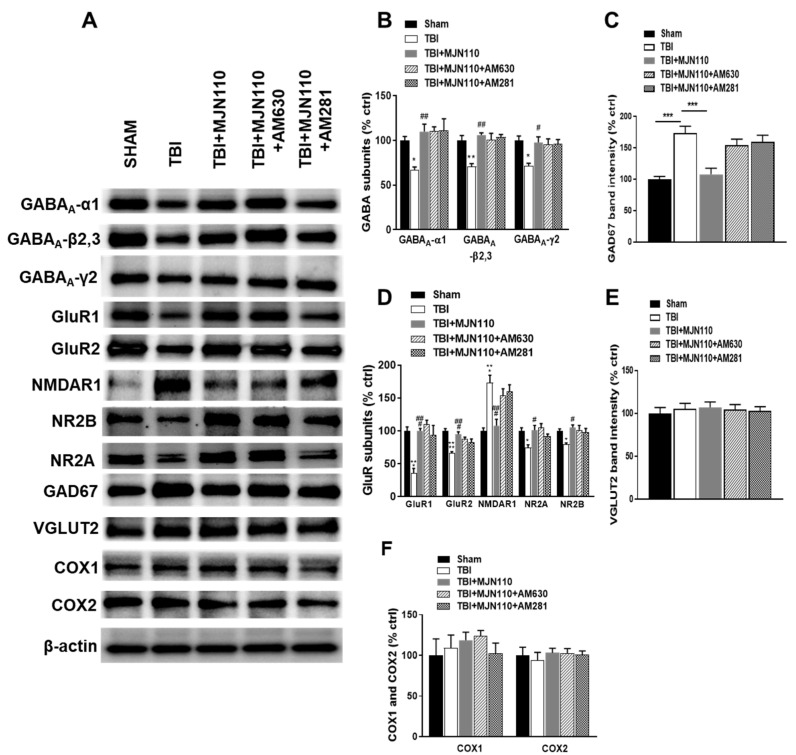
The reversed expression of glutamate and GABA_A_ receptor subunits by MJN110 was independent of cannabinoid receptor signaling. (**A**–**F**) Western blot analysis of hippocampal GABA_A_-α1, GABA_A_-β 2,3, GABA_A_-γ2, GluR1, GluR2, NMDAR1, NR2A, NR2B, GAD67, VGLUT2, COX1 and COX2 at 30 days post-TBI. (* *p* < 0.05, ** *p* < 0.01 and *** *p* < 0.001 comparing sham vs. TBI/vehicle groups; # *p* < 0.05, ## *p* < 0.01 and ### *p* < 0.001 comparing TBI vs. TBI + MJN110 (2.5 mg/kg) groups). *n* = 5 animals in each group; Bars represent means ± SEM.

To determine whether alteration of the glutamate and GABA receptor components is persistent and thus contributes to the long-term memory and cognitive deficits [35,36], the expression of the NMDA, AMPA and GABA receptors was also examined at 30 days post-TBI. Similar to the findings at 8 days post-TBI, the hippocampal expression of GABA_A_ -α1, -β2,3 and -γ2, GluR1, GluR2, NR2A, NR2B at 30 days post-TBI was also significantly reduced compared to the sham control animals, and all the changes were restored in the MJN110 (2.5 mg/kg) treatment group (Figure 8A,B,D). Conversely, the expression of the NMDAR1 subunit (Figure 8D) and GAD67 (Figure 8C) was dramatically increased in the TBI group and reduced by MJN110 treatment. There were no changes in the expression of VGLUT2, COX1 and COX2 in all the experimental groups (Figure 8E,F). Co-treatment with AM281 or AM630 did not alter the effect of MJN110 on the expression of these receptors. 

### 3.7. The Therapeutic Effects of MJN110 Were Eliminated by Simultaneous Blockade of 2-AG Synthesis in the TBI Mouse Brain

**Figure 9:**
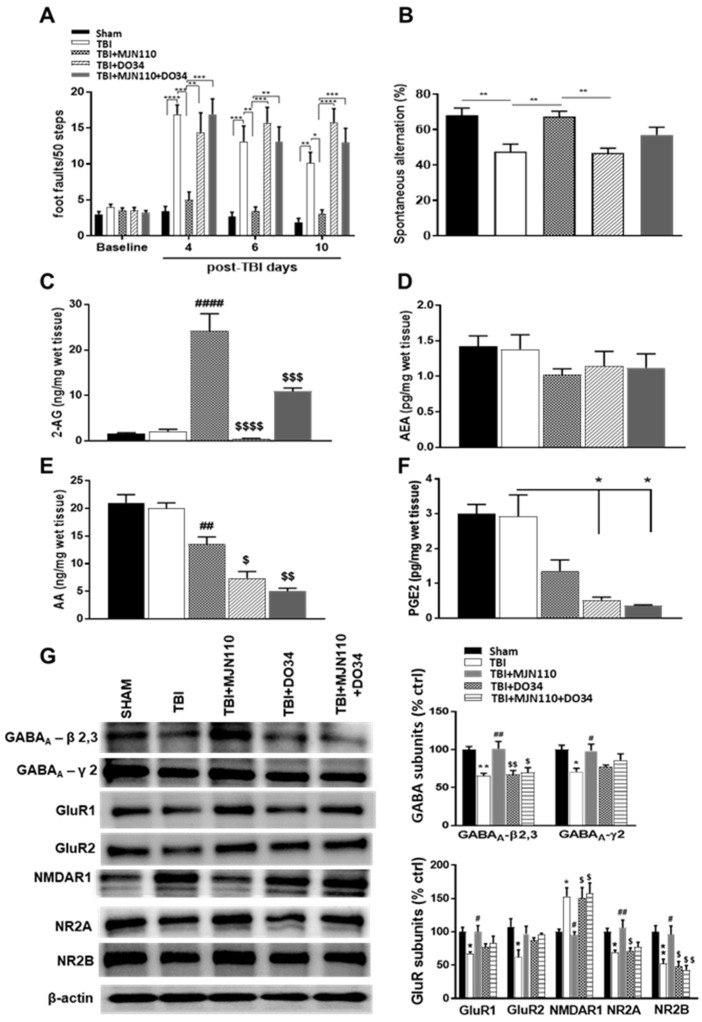
Improvement on locomotor function, working memory and the expression of synaptic components by MJN110 was reversed by blockade of 2-AG synthesis. (**A**) Beam walk test was conducted at 4, 6 and 10 days post-TBI (* *p* < 0.05, ** *p* < 0.01, *** *p* < 0.001 and **** *p* < 0.001). (**B**) On day 13 post injury, spontaneous alternation Y maze test was performed (** *p* < 0.01). (**C**–**E**) Ipsilateral cortical tissues on day 4 post-TBI were subjected to LC-MS/MS analysis to measure 2-AG, AEA and AA. (## *p* < 0.01 and #### *p* < 0.001 comparing TBI vs. MJN110 (1 mg/kg); $ *p* < 0.05, $$ *p* < 0.01, $$$ *p* < 0.001 and $$$$ *p* < 0.001 comparing MJN110 (1 mg/kg) vs. DO34 (30 mg/kg) or DO34 + MJN110). (**F**) The ipsilateral cortical tissues at day 4 post-TBI were assessed for PGE_2_ production by ELISA. (* *p* < 0.05 comparing TBI vs. DO34 or DO34 + MJN110). (**G**) Western blot analysis of the hippocampal GABA_A_-β2,3, GABA_A_-γ2, GluR1, GluR2, NMDAR1, NR2A and NR2B expression at 14 days post-TBI (* *p* < 0.05 and ** *p* < 0.01 comparing sham vs. TBI; # *p* < 0.05 and ## *p* < 0.01 comparing TBI vs. TBI + MJN110; $ *p* < 0.05 and $$ *p* < 0.01 comparing MJN110 vs. DO34 and DO34 + MJN110). *n* = 5–10 animals in each group; Bars represent means ± SEM.

To further validate the therapeutic effect of MJN110 is attributable to the elevated brain levels of 2-AG, we utilized a recently developed DAGL inhibitor, DO34, to block the 2-AG synthesis [29]. TBI animals were treated with DO34 (30 mg/kg), MJN110 (1 mg/kg) and their combination once a day for 7 days. Inhibition of DAGLα and DAGLβ by DO34 reversed the therapeutic effect of MJN110 on locomotor function and working memory in both beam walk and Y-maze tests, whereas treatment with DO34 alone did not have any effect (Figure 9A,B). To assess the selectivity of systematically administered DO34 in the mouse brain, 2-AG, AEA and AA levels were measured in the ipsilateral cortex at 4 days post-TBI using LC-MS/MS. We found that DO34 treatment almost completely blocked the synthesis of 2-AG and the elevated levels of 2-AG by MJN110 were significantly reduced (Figure 9C). Notably, there was no change in the levels of AEA among the groups (Figure 9D). TBI per se did not affect the levels of AA compared to the sham group, but treatment with DO34 or its combination with MJN110 significantly reduced AA levels (Figure 9E). The levels of PGE_2_ in the TBI mouse brain was also significantly reduced by DO34 and its combined treatment with MJN110 (Figure 9F). Treatment with MJN110 also normalized the expression of GABA_A_ -β2, 3, GABA_A_ -γ2, GluR1, GluR2, NR2A, NR2B and NMDAR1 receptor subunits in hippocampal tissue lysates at 14 days post-TBI and these changes were mostly reversed by co-treatment with DO34. Treatment with DO34 alone did not have any effect on the altered expression of these inotropic receptor subunits in the TBI mouse hippocampus (Figure 9G).

## 4. Discussion

Accumulating evidence supports that augmentation of endocannabinoids appears to be an effective strategy for the treatment of many neurological and neuropsychiatric diseases [6,7]. However, the underlying therapeutic mechanisms remain unclear. In this study, we found that selective inhibition of MAGL with MJN110 elevated the brain levels of 2-AG and reduced the production of AA and PGE_2_. Treatment with MJN110 suppressed neuroinflammation, normalized the expression of glutamate and GABA receptor subunits and improved motor and cognitive function. The improvement in locomotor function and working memory in the TBI animals seems to be mediated by activation of cannabinoid receptors. Furthermore, we found that the therapeutic effect of MJN110 was diminished when the 2-AG synthetic pathway was blocked suggesting that the production of 2-AG is critically involved in neuroprotection. 

Neuroinflammation immediately following TBI is a major stepping-stone to initiate the pathological processes and may cause long-term neurological deficits and behavioral abnormalities [33,37]. Numerous studies have shown that TBI is accompanied by infiltration of peripheral immune cells and activation of resident glial cells in the brain through cytokine and chemokine signaling, and the release of many inflammatory mediators [38]. Consistently, we observed the accumulation of astrocytes and microglia and the increased production of pro-inflammatory mediators IL-6, iNOS, IL-1β, TNF-α and COX-1 in the ipsilateral cerebral cortex in our repetitive mTBI mouse model. Neuronal cell death was also observed in the cortex and hippocampal dentate gyrus of the TBI mouse brain. We and others have previously shown that enhancement of ERK and AKT signaling may improve motor and cognitive function after TBI [13,18,39,40]. Here we found that inhibition of MAGL increased the brain levels of 2-AG and reduced the production of pro-inflammatory mediators and neuronal cell death, possibly through activation of ERK and AKT, two widely studied pathways known for their roles promoting survival and improving neurological outcomes after injury to the brain and spinal cord [13,14,30,41]. Despite that 2-AG can exert its beneficial effects via cannabinoid receptors [38], the involvement of CB1 and CB2 receptors in the action of the inhibitors of 2-AG hydrolytic enzymes remains uncertain. Using the CB1 and CB2 receptor antagonists, we found that the locomotor function recovery is mediated by both CB1 and CB2 receptors in the first several days following brain injury, and that activation of CB1 receptor contributes to working memory recovery at the relatively late time point. However, the improvement of MJN110 treatment on TBI-induced spatial learning and memory deficits is independent on cannabinoid receptors. Although co-administration of the CB1 receptor antagonist tends to reverse the improved performance of MJN110 in both the Morris water maze acquisition phase and the subsequent probe trial, there were no statistically significant differences in the MJN110 alone and MJN110 plus AM281 treatment groups. These findings are in agreement with the study using the experimental ischemic stroke model treated with the MAGL inhibitor JZL184 [27]. These results indicate that the behavioral improvements of MAGL inhibition in this TBI mouse model are attributable to both cannabinoid receptor dependent and independent mechanisms.

Neuronal cell death following brain injury could directly or indirectly influence synaptic plasticity, neuronal circuitry and signaling transmission [42,43,44]. Our recent study revealed that the reduced expression of several SNARE proteins including synaptosome-associated protein of 25 kDa (SNAP25), and cysteine string protein alpha (α-CSP) at one month post-injury is associated with TBI induced cognitive deficits [18]. Maintenance of proper synaptic integrity is crucial for brain development, synaptic function as well as mitigation of neuropathology [45,46,47]. Exaggerated activation of NMDA receptors causes deregulated Ca^++^ influx and neuronal cell death in stroke, ischemia, TBI and other neurodegenerative diseases such as Alzheimer’s, Parkinson’s and Huntington’s diseases [35,48]. NMDA receptor activation in hippocampal neurons controls the induction of long-term potentiation (LTP) which is associated with memory formation [49,50]. Consistent with previous reports in the mild repetitive closed head injury mouse model [15,51], our current study using hippocampal tissue lysates from 8, 14 and 30 days post-TBI revealed a reduced expression of NMDA receptor subunits (NR2A, NR2B), and the AMPA receptor subunits (GluR1, GluR2), and these changes might contribute to the reduction of LTP and the impaired cognitive function. 

Examination of the postmortem brain tissues from Alzheimer’s patients showed a decrease in the expression of NR2A and NR2B mRNA and protein levels in the hippocampus and entorhinal cortex [52,53]. Deletion of the NR2A subunit of the NMDA receptors resulted in significant reduction of hippocampal LTP and spatial learning deficits [54], whereas overexpression of the NR2B receptor subunits enhanced synaptic potentiation as well as learning and memory [55]. The reduced expression of NR2A and NR2B in our current TBI mouse model might be due to the cleavage of their intracellular c-termini by the increased activity of calpain, and thus resulting in the uncoupling of the NMDA receptors from downstream survival pathways as reported in the animal model of focal cerebral ischemia [56]. Despite the expression of both NR2A and NR2B was reduced, it is likely that these NMDA receptor subunits could be differentially regulated. The increased ratio of the NR2A and NR2B subunits was demonstrated to cause long-term memory deficits [57], suggesting that the proper balance between these two subunits is also critical for the maintenance of normal synaptic neurotransmission. Given that NR2A and NR2B are the dominant NMDA receptor components expressed at the respective synaptic and extrasynaptic sites, the composition and function of both synaptic and extrasynaptic NMDA receptors are likely to be impaired following repetitive TBI, and thus are attributable to the reduced cognitive function. It has been reported that AMPA receptor subunits can control the trafficking and delivery of NMDA receptors to the plasma membrane and synaptic compartments through their intercellular domains [58]. Reduced GluR2 receptor expression in the glutamate tetrameric receptor complex was linked to excitotoxicity [59] and found in several neurological diseases like ischemia [60,61], epilepsy [62,63] and brain trauma [64]. Studies in schizophrenia patients and Parkinson’s disease animal models also revealed that alteration of NMDA receptor trafficking, and its decreased expression might contribute to the cognition and behavioral deficits [15,65,66]. All these studies suggest that LTP dependent memory formation and recognition is dependent on synaptic strength and plasticity which is functionally maintained by the NMDA and AMPA receptors at the synaptic terminals. The improvement of MJN110 on neurological and behavioral deficits is likely attributable to the restoration of TBI-induced loss of hippocampal NMDA and AMPA receptor subunits.

Another important synaptic components are GABA_A_ receptors, that are essential for GABAergic stimulation and the synchronization of oscillatory activities required for the normal cognition [67]. Several studies in schizophrenia patients found an altered expression of GABA_A_ receptor subunits and GABA synthesis in the cerebral cortex, and electrophysiological dysfunction in parvalbumin and somatostatin GABAergic interneurons in the cortex and hippocampus [68,69,70]. Blockade of the fast inhibitory GABA_A_ receptors by bicuculline, pentylenetetrazol or picrotoxin causes severe motor seizures in experimental animals [71]. A rat controlled cortical impact-induced TBI model study showed a long-lasting working memory impairment associated with increased levels of GAD67 one month after injury [72]. The loss of GABAergic interneurons and the reduced surface expression of GABA_A_ receptors (GABA_A_ -α1, -β2 and -γ2) in hippocampus and amygdala of TBI animals lead to epileptic seizure and long-lasting anxiety disorders [36,73,74,75,76,77]. Our current study demonstrated that the expression of GABA_A_ -α1, -β2, 3 and -γ2 receptor subunits in the ipsilateral hippocampus was reduced at 8, 14 and 30 days post-TBI, and treatment with MJN110 normalized the expression of GABA_A_ receptor subunits in hippocampus. Among the GABA_A_ β receptor subunits, β3 is shown to play a crucial role in GABAergic inhibitory transmission [78]. Moreover, a recent study found that 2-AG can interact with GABA_A_ β2 receptor subunit in the presence of low concentration of GABA to modulate locomotion behavior [79]. Taken together, our results suggest that MAGL inhibition can benefit TBI treatment through elevation of the endogenous 2-AG levels which can restore the dysregulated excitatory and inhibitory neurotransmission and maintain the homeostasis of neuronal signaling.

The endogenous levels of 2-AG are tightly regulated by its synthesis and degradation. 2-AG in brain is chiefly synthesized by DAGLα, and to a lesser extent, by DAGLβ. Pharmacological inhibition or targeted gene deletion of DAGLα lowers brain levels of 2-AG, impairs synaptic plasticity, hippocampal dependent learning and memory, fear extinction and triggers trauma-like behaviors [80,81,82,83]. However, recent studies demonstrated that DAGLβ is the major enzyme for 2-AG synthesis in microglia/macrophage, and genetic deletion of DAGLβ suppressed inflammatory and neuropathic pain [84,85,86]. Consistently, the dual DAGLα and DAGLβ inhibitor DO34 reversed LPS-induced allodynia in mice with DAGLα deletion but had no effect on the antinociceptive effect of DAGLβ knockout mice [87]. Interestingly, a recent study showed that DAGLβ knockout male mice are resistant to TBI-induced mortality, but had no effect on motor and cognitive function, suggesting that blockade of AA and the subsequent prostaglandin production in reactive microglia/macrophages by targeting DAGLβ is unable to exert neuroprotective effects [88]. In our current study, treatment with DO34 significantly reduced the brain levels of 2-AG, AA and PGE_2_, and reversed the MJN110 mediated beneficial effects in the TBI animals. These results suggest that the production of 2-AG rather than the reduced eicosanoid signaling is imperative for the therapeutic effects of the MAGL inhibitors.

## Data Availability

The data presented in this study will be made available upon reasonable request.

## References

[1] Reilly P. (2007). The impact of neurotrauma on society: An international perspective. Prog. Brain Res..

[2] Faul M., Coronado V. (2015). Epidemiology of traumatic brain injury. Handb. Clin. Neurol..

[3] Hobbs J.G., Young J.S., Bailes J.E. (2016). Sports-related concussions: Diagnosis, complications, and current management strategies. Neurosurg. Focus.

[4] Lindberg M.A., Kiser S.A., Moy Martin E.M. (2020). Mild TBI/Concussion Clinical Tools for Providers Used Within the Department of Defense and Defense Health Agency. Fed. Pract..

[5] Johnson V.E., Stewart W., Arena J.D., Smith D.H. (2017). Traumatic Brain Injury as a Trigger of Neurodegeneration. Adv. Neurobiol..

[6] Pertwee R.G. (2014). Elevating endocannabinoid levels: Pharmacological strategies and potential therapeutic applications. Proc. Nutr. Soc..

[7] Hill M.N., Patel S. (2013). Translational evidence for the involvement of the endocannabinoid system in stress-related psychiatric illnesses. Biol. Mood Anxiety Disord..

[8] Piomelli D. (2003). The molecular logic of endocannabinoid signalling. Nat. Rev. Neurosci..

[9] Mackie K. (2006). Cannabinoid receptors as therapeutic targets. Annu. Rev. Pharmacol. Toxicol..

[10] Di Marzo V. (2009). The endocannabinoid system: Its general strategy of action, tools for its pharmacological manipulation and potential therapeutic exploitation. Pharmacol. Res..

[11] Hwang J., Adamson C., Butler D., Janero D.R., Makriyannis A., Bahr B.A. (2010). Enhancement of endocannabinoid signaling by fatty acid amide hydrolase inhibition: A neuroprotective therapeutic modality. Life Sci..

[12] Ueda N., Tsuboi K., Uyama T., Ohnishi T. (2011). Biosynthesis and degradation of the endocannabinoid 2-arachidonoylglycerol. Biofactors.

[13] Tchantchou F., Tucker L.B., Fu A.H., Bluett R.J., McCabe J.T., Patel S., Zhang Y. (2014). The fatty acid amide hydrolase inhibitor PF-3845 promotes neuronal survival, attenuates inflammation and improves functional recovery in mice with traumatic brain injury. Neuropharmacology.

[14] Tchantchou F., Zhang Y. (2013). Selective inhibition of alpha/beta-hydrolase domain 6 attenuates neurodegeneration, alleviates blood brain barrier breakdown, and improves functional recovery in a mouse model of traumatic brain injury. J. Neurotrauma.

[15] Zhang J., Teng Z., Song Y., Hu M., Chen C. (2015). Inhibition of monoacylglycerol lipase prevents chronic traumatic encephalopathy-like neuropathology in a mouse model of repetitive mild closed head injury. J. Cereb. Blood Flow Metab..

[16] Katz P.S., Sulzer J.K., Impastato R.A., Teng S.X., Rogers E.K., Molina P.E. (2015). Endocannabinoid degradation inhibition improves neurobehavioral function, blood-brain barrier integrity, and neuroinflammation following mild traumatic brain injury. J. Neurotrauma.

[17] Mayeux J., Katz P., Edwards S., Middleton J.W., Molina P.E. (2017). Inhibition of Endocannabinoid Degradation Improves Outcomes from Mild Traumatic Brain Injury: A Mechanistic Role for Synaptic Hyperexcitability. J. Neurotrauma.

[18] Selvaraj P., Wen J., Tanaka M., Zhang Y. (2019). Therapeutic Effect of a Novel Fatty Acid Amide Hydrolase Inhibitor PF04457845 in the Repetitive Closed Head Injury Mouse Model. J. Neurotrauma.

[19] Schurman L.D., Lichtman A.H. (2017). Endocannabinoids: A Promising Impact for Traumatic Brain Injury. Front. Pharmacol..

[20] Panikashvili D., Simeonidou C., Ben-Shabat S., Hanus L., Breuer A., Mechoulam R., Shohami E. (2001). An endogenous cannabinoid (2-AG) is neuroprotective after brain injury. Nature.

[21] Panikashvili D., Shein N.A., Mechoulam R., Trembovler V., Kohen R., Alexandrovich A., Shohami E. (2006). The endocannabinoid 2-AG protects the blood-brain barrier after closed head injury and inhibits mRNA expression of proinflammatory cytokines. Neurobiol. Dis..

[22] Shohami E., Cohen-Yeshurun A., Magid L., Algali M., Mechoulam R. (2011). Endocannabinoids and traumatic brain injury. Br. J. Pharmacol..

[23] Nomura D.K., Morrison B.E., Blankman J.L., Long J.Z., Kinsey S.G., Marcondes M.C., Ward A.M., Hahn Y.K., Lichtman A.H., Conti B. (2011). Endocannabinoid hydrolysis generates brain prostaglandins that promote neuroinflammation. Science.

[24] Niphakis M.J., Cognetta A.B., Chang J.W., Buczynski M.W., Parsons L.H., Byrne F., Burston J.J., Chapman V., Cravatt B.F. (2013). Evaluation of NHS carbamates as a potent and selective class of endocannabinoid hydrolase inhibitors. ACS Chem. Neurosci..

[25] Ignatowska-Jankowska B., Wilkerson J.L., Mustafa M., Abdullah R., Niphakis M., Wiley J.L., Cravatt B.F., Lichtman A.H. (2015). Selective monoacylglycerol lipase inhibitors: Antinociceptive versus cannabimimetic effects in mice. J. Pharmacol. Exp. Ther..

[26] Serrano A., Pavon F.J., Buczynski M.W., Schlosburg J., Natividad L.A., Polis I.Y., Stouffer D.G., Zorrilla E.P., Roberto M., Cravatt B.F. (2018). Deficient endocannabinoid signaling in the central amygdala contributes to alcohol dependence-related anxiety-like behavior and excessive alcohol intake. Neuropsychopharmacology.

[27] Choi S.H., Arai A.L., Mou Y., Kang B., Yen C.C., Hallenbeck J., Silva A.C. (2018). Neuroprotective Effects of MAGL (Monoacylglycerol Lipase) Inhibitors in Experimental Ischemic Stroke. Stroke.

[28] Fucich E.A., Stielper Z.F., Cancienne H.L., Edwards S., Gilpin N.W., Molina P.E., Middleton J.W. (2020). Endocannabinoid degradation inhibitors ameliorate neuronal and synaptic alterations following traumatic brain injury. J. Neurophysiol..

[29] Ogasawara D., Deng H., Viader A., Baggelaar M.P., Breman A., den Dulk H., van den Nieuwendijk A.M., Soethoudt M., van der Wel T., Zhou J. (2016). Rapid and profound rewiring of brain lipid signaling networks by acute diacylglycerol lipase inhibition. Proc. Natl. Acad. Sci. USA.

[30] Yu F., Wang Z., Tchantchou F., Chiu C.T., Zhang Y., Chuang D.M. (2012). Lithium ameliorates neurodegeneration, suppresses neuroinflammation, and improves behavioral performance in a mouse model of traumatic brain injury. J. Neurotrauma.

[31] Velosky A.G., Tucker L.B., Fu A.H., Liu J., McCabe J.T. (2017). Cognitive performance of male and female C57BL/6J mice after repetitive concussive brain injuries. Behav. Brain Res..

[32] Tanaka K., Yagi T., Shimakoshi R., Azuma K., Nanba T., Ogo H., Tamura A., Asanuma M. (2009). Effects of galantamine on L-NAME-induced behavioral impairment in Y-maze task in mice. Neurosci. Lett..

[33] Jassam Y.N., Izzy S., Whalen M., McGavern D.B., El Khoury J. (2017). Neuroimmunology of Traumatic Brain Injury: Time for a Paradigm Shift. Neuron.

[34] Pearn M.L., Niesman I.R., Egawa J., Sawada A., Almenar-Queralt A., Shah S.B., Duckworth J.L., Head B.P. (2017). Pathophysiology Associated with Traumatic Brain Injury: Current Treatments and Potential Novel Therapeutics. Cell Mol. Neurobiol..

[35] Carvajal F.J., Mattison H.A., Cerpa W. (2016). Role of NMDA Receptor-Mediated Glutamatergic Signaling in Chronic and Acute Neuropathologies. Neural Plast..

[36] Wu C., Sun D. (2015). GABA receptors in brain development, function, and injury. Metab. Brain Dis..

[37] Simon D.W., McGeachy M.J., Bayir H., Clark R.S., Loane D.J., Kochanek P.M. (2017). The far-reaching scope of neuroinflammation after traumatic brain injury. Nat. Rev. Neurol..

[38] Mechoulam R., Spatz M., Shohami E. (2002). Endocannabinoids and neuroprotection. Sci. STKE.

[39] Dash P.K., Mach S.A., Moore A.N. (2002). The role of extracellular signal-regulated kinase in cognitive and motor deficits following experimental traumatic brain injury. Neuroscience.

[40] Zhang C., Zhu J., Zhang J., Li H., Zhao Z., Liao Y., Wang X., Su J., Sang S., Yuan X. (2014). Neuroprotective and anti-apoptotic effects of valproic acid on adult rat cerebral cortex through ERK and Akt signaling pathway at acute phase of traumatic brain injury. Brain Res..

[41] Walker C.L., Liu N.K., Xu X.M. (2013). PTEN/PI3K and MAPK signaling in protection and pathology following CNS injuries. Front. Biol..

[42] Sato M., Chang E., Igarashi T., Noble L.J. (2001). Neuronal injury and loss after traumatic brain injury: Time course and regional variability. Brain Res..

[43] Hemphill M.A., Dauth S., Yu C.J., Dabiri B.E., Parker K.K. (2015). Traumatic brain injury and the neuronal microenvironment: A potential role for neuropathological mechanotransduction. Neuron.

[44] Gao X., Chen J. (2011). Mild traumatic brain injury results in extensive neuronal degeneration in the cerebral cortex. J. Neuropathol. Exp. Neurol..

[45] Barria A., Malinow R. (2005). NMDA receptor subunit composition controls synaptic plasticity by regulating binding to CaMKII. Neuron.

[46] Gardoni F., Polli F., Cattabeni F., Di Luca M. (2006). Calcium-calmodulin-dependent protein kinase II phosphorylation modulates PSD-95 binding to NMDA receptors. Eur. J. Neurosci..

[47] Fan X., Jin W.Y., Wang Y.T. (2014). The NMDA receptor complex: A multifunctional machine at the glutamatergic synapse. Front. Cell. Neurosci..

[48] Hynd M.R., Scott H.L., Dodd P.R. (2004). Glutamate-mediated excitotoxicity and neurodegeneration in Alzheimer’s disease. Neurochem. Int..

[49] Bliss T.V., Collingridge G.L. (1993). A synaptic model of memory: Long-term potentiation in the hippocampus. Nature.

[50] Collingridge G.L., Bliss T.V. (1995). Memories of NMDA receptors and LTP. Trends Neurosci..

[51] Mannix R., Berkner J., Mei Z., Alcon S., Hashim J., Robinson S., Jantzie L., Meehan W.P., Qiu J. (2017). Adolescent Mice Demonstrate a Distinct Pattern of Injury after Repetitive Mild Traumatic Brain Injury. J. Neurotrauma.

[52] Sze C., Bi H., Kleinschmidt-DeMasters B.K., Filley C.M., Martin L.J. (2001). *N*-Methyl-D-aspartate receptor subunit proteins and their phosphorylation status are altered selectively in Alzheimer’s disease. J. Neurol. Sci..

[53] Hynd M.R., Scott H.L., Dodd P.R. (2004). Differential expression of *N*-methyl-d-aspartate receptor NR2 isoforms in Alzheimer’s disease. J. Neurochem..

[54] Sakimura K., Kutsuwada T., Ito I., Manabe T., Takayama C., Kushiya E., Yagi T., Aizawa S., Inoue Y., Sugiyama H. (1995). Reduced hippocampal LTP and spatial learning in mice lacking NMDA receptor epsilon 1 subunit. Nature.

[55] Tang Y.P., Shimizu E., Dube G.R., Rampon C., Kerchner G.A., Zhuo M., Liu G., Tsien J.Z. (1999). Genetic enhancement of learning and memory in mice. Nature.

[56] Gascon S., Sobrado M., Roda J.M., Rodriguez-Pena A., Diaz-Guerra M. (2008). Excitotoxicity and focal cerebral ischemia induce truncation of the NR2A and NR2B subunits of the NMDA receptor and cleavage of the scaffolding protein PSD-95. Mol. Psychiatry.

[57] Cui Z., Feng R., Jacobs S., Duan Y., Wang H., Cao X., Tsien J.Z. (2013). Increased NR2A:NR2B ratio compresses long-term depression range and constrains long-term memory. Sci. Rep..

[58] Traynelis S.F., Wollmuth L.P., McBain C.J., Menniti F.S., Vance K.M., Ogden K.K., Hansen K.B., Yuan H., Myers S.J., Dingledine R. (2010). Glutamate receptor ion channels: Structure, regulation, and function. Pharmacol. Rev..

[59] Kwak S., Weiss J.H. (2006). Calcium-permeable AMPA channels in neurodegenerative disease and ischemia. Curr. Opin. Neurobiol..

[60] Liu B., Liao M., Mielke J.G., Ning K., Chen Y., Li L., El-Hayek Y.H., Gomez E., Zukin R.S., Fehlings M.G. (2006). Ischemic insults direct glutamate receptor subunit 2-lacking AMPA receptors to synaptic sites. J. Neurosci..

[61] Noh K.M., Yokota H., Mashiko T., Castillo P.E., Zukin R.S., Bennett M.V. (2005). Blockade of calcium-permeable AMPA receptors protects hippocampal neurons against global ischemia-induced death. Proc. Natl. Acad. Sci. USA.

[62] Rakhade S.N., Zhou C., Aujla P.K., Fishman R., Sucher N.J., Jensen F.E. (2008). Early alterations of AMPA receptors mediate synaptic potentiation induced by neonatal seizures. J. Neurosci..

[63] Bell J.D., Ai J., Chen Y., Baker A.J. (2007). Mild in vitro trauma induces rapid Glur2 endocytosis, robustly augments calcium permeability and enhances susceptibility to secondary excitotoxic insult in cultured Purkinje cells. Brain.

[64] Spaethling J.M., Klein D.M., Singh P., Meaney D.F. (2008). Calcium-permeable AMPA receptors appear in cortical neurons after traumatic mechanical injury and contribute to neuronal fate. J. Neurotrauma.

[65] Noga J.T., Hyde T.M., Herman M.M., Spurney C.F., Bigelow L.B., Weinberger D.R., Kleinman J.E. (1997). Glutamate receptors in the postmortem striatum of schizophrenic, suicide, and control brains. Synapse.

[66] Sokolov B.P. (1998). Expression of NMDAR1, GluR1, GluR7, and KA1 glutamate receptor mRNAs is decreased in frontal cortex of “neuroleptic-free” schizophrenics: Evidence on reversible up-regulation by typical neuroleptics. J. Neurochem..

[67] Klausberger T., Somogyi P. (2008). Neuronal diversity and temporal dynamics: The unity of hippocampal circuit operations. Science.

[68] Lewis D.A., Cho R.Y., Carter C.S., Eklund K., Forster S., Kelly M.A., Montrose D. (2008). Subunit-selective modulation of GABA type A receptor neurotransmission and cognition in schizophrenia. Am. J. Psychiatry.

[69] Benes F.M., Berretta S. (2001). GABAergic interneurons: Implications for understanding schizophrenia and bipolar disorder. Neuropsychopharmacology.

[70] Zhang Z.J., Reynolds G.P. (2002). A selective decrease in the relative density of parvalbumin-immunoreactive neurons in the hippocampus in schizophrenia. Schizophr. Res..

[71] Sperk G., Furtinger S., Schwarzer C., Pirker S. (2004). GABA and its receptors in epilepsy. Adv. Exp. Med. Biol..

[72] Kobori N., Dash P.K. (2006). Reversal of brain injury-induced prefrontal glutamic acid decarboxylase expression and working memory deficits by D1 receptor antagonism. J. Neurosci..

[73] Almeida-Suhett C.P., Prager E.M., Pidoplichko V., Figueiredo T.H., Marini A.M., Li Z., Eiden L.E., Braga M.F. (2014). Reduced GABAergic inhibition in the basolateral amygdala and the development of anxiety-like behaviors after mild traumatic brain injury. PLoS ONE.

[74] Almeida-Suhett C.P., Prager E.M., Pidoplichko V., Figueiredo T.H., Marini A.M., Li Z., Eiden L.E., Braga M.F. (2015). GABAergic interneuronal loss and reduced inhibitory synaptic transmission in the hippocampal CA1 region after mild traumatic brain injury. Exp. Neurol..

[75] Gibson C.J., Meyer R.C., Hamm R.J. (2010). Traumatic brain injury and the effects of diazepam, diltiazem, and MK-801 on GABA-A receptor subunit expression in rat hippocampus. J. Biomed. Sci..

[76] Lopez-Picon F., Snellman A., Shatillo O., Lehtiniemi P., Gronroos T.J., Marjamaki P., Trigg W., Jones P.A., Solin O., Pitkanen A. (2016). Ex Vivo Tracing of NMDA and GABA-A Receptors in Rat Brain After Traumatic Brain Injury Using 18F-GE-179 and 18F-GE-194 Autoradiography. J. Nucl. Med..

[77] Drexel M., Puhakka N., Kirchmair E., Hortnagl H., Pitkanen A., Sperk G. (2015). Expression of GABA receptor subunits in the hippocampus and thalamus after experimental traumatic brain injury. Neuropharmacology.

[78] Nguyen Q.A., Nicoll R.A. (2018). The GABAA Receptor beta Subunit Is Required for Inhibitory Transmission. Neuron.

[79] Sigel E., Baur R., Racz I., Marazzi J., Smart T.G., Zimmer A., Gertsch J. (2011). The major central endocannabinoid directly acts at GABA(A) receptors. Proc. Natl. Acad. Sci. USA.

[80] Gao Y., Vasilyev D.V., Goncalves M.B., Howell F.V., Hobbs C., Reisenberg M., Shen R., Zhang M.Y., Strassle B.W., Lu P. (2010). Loss of retrograde endocannabinoid signaling and reduced adult neurogenesis in diacylglycerol lipase knock-out mice. J. Neurosci..

[81] Tanimura A., Yamazaki M., Hashimotodani Y., Uchigashima M., Kawata S., Abe M., Kita Y., Hashimoto K., Shimizu T., Watanabe M. (2010). The endocannabinoid 2-arachidonoylglycerol produced by diacylglycerol lipase alpha mediates retrograde suppression of synaptic transmission. Neuron.

[82] Cavener V.S., Gaulden A., Pennipede D., Jagasia P., Uddin J., Marnett L.J., Patel S. (2018). Inhibition of Diacylglycerol Lipase Impairs Fear Extinction in Mice. Front. Neurosci..

[83] Schurman L.D., Carper M.C., Moncayo L.V., Ogasawara D., Richardson K., Yu L., Liu X., Poklis J.L., Liu Q.S., Cravatt B.F. (2019). Diacylglycerol Lipase-Alpha Regulates Hippocampal-Dependent Learning and Memory Processes in Mice. J. Neurosci..

[84] Hsu K.L., Tsuboi K., Adibekian A., Pugh H., Masuda K., Cravatt B.F. (2012). DAGLbeta inhibition perturbs a lipid network involved in macrophage inflammatory responses. Nat. Chem. Biol..

[85] Viader A., Ogasawara D., Joslyn C.M., Sanchez-Alavez M., Mori S., Nguyen W., Conti B., Cravatt B.F. (2016). A chemical proteomic atlas of brain serine hydrolases identifies cell type-specific pathways regulating neuroinflammation. Elife.

[86] Wilkerson J.L., Ghosh S., Bagdas D., Mason B.L., Crowe M.S., Hsu K.L., Wise L.E., Kinsey S.G., Damaj M.I., Cravatt B.F. (2016). Diacylglycerol lipase beta inhibition reverses nociceptive behaviour in mouse models of inflammatory and neuropathic pain. Br. J. Pharmacol..

[87] Wilkerson J.L., Donvito G., Grim T.W., Abdullah R.A., Ogasawara D., Cravatt B.F., Lichtman A.H. (2017). Investigation of Diacylglycerol Lipase Alpha Inhibition in the Mouse Lipopolysaccharide Inflammatory Pain Model. J. Pharmacol. Exp. Ther..

[88] O’Brien L.D., Smith T.L., Donvito G., Cravatt B.F., Newton J., Spiegel S., Reeves T.M., Phillips L.L., Lichtman A.H. (2021). Diacylglycerol Lipase-beta Knockout Mice Display a Sex-Dependent Attenuation of Traumatic Brain Injury-Induced Mortality with No Impact on Memory or Other Functional Consequences. Cannabis Cannabinoid Res..

